# A Feasible Alternative to FDSOI and FinFET: Optimization of W/La_2_O_3_/Si Planar PMOS with 14 nm Gate-Length

**DOI:** 10.3390/ma14195721

**Published:** 2021-09-30

**Authors:** Siew Kien Mah, Pin Jern Ker, Ibrahim Ahmad, Noor Faizah Zainul Abidin, Mansur Mohammed Ali Gamel

**Affiliations:** 1 Institute of Sustainable Energy, Universiti Tenaga Nasional, Kajang 43000, Malaysia; PinJern@uniten.edu.my (P.J.K.); AIbrahim@uniten.edu.my (I.A.); noorfaizah.zaha@gmail.com (N.F.Z.A.); mansurgamel@gmail.com (M.M.A.G.); 2 Department of Electrical Engineering, Nilai University, Nilai 71800, Malaysia

**Keywords:** MOSFET, FDSOI, FinFET, Taguchi, PMOS

## Abstract

At the 90-nm node, the rate of transistor miniaturization slows down due to challenges in overcoming the increased leakage current (*I_off_*). The invention of high-k/metal gate technology at the 45-nm technology node was an enormous step forward in extending Moore’s Law. The need to satisfy performance requirements and to overcome the limitations of planar bulk transistor to scales below 22 nm led to the development of fully depleted silicon-on-insulator (FDSOI) and fin field-effect transistor (FinFET) technologies. The 28-nm wafer planar process is the most cost-effective, and scaling towards the sub-10 nm technology node involves the complex integration of new materials (Ge, III-V, graphene) and new device architectures. To date, planar transistors still command >50% of the transistor market and applications. This work aims to downscale a planar PMOS to a 14-nm gate length using La_2_O_3_ as the high-k dielectric material. The device was virtually fabricated and electrically characterized using SILVACO. Taguchi L9 and L27 were employed to study the process parameters’ variability and interaction effects to optimize the process parameters to achieve the required output. The results obtained from simulation using the SILVACO tool show good agreement with the nominal values of PMOS threshold voltage (*V_th_*) of −0.289 V ± 12.7% and *I_off_* of less than 10^−7^ A/µm, as projected by the International Technology Roadmap for Semiconductors (ITRS). Careful control of SiO_2_ formation at the Si interface and rapid annealing processing are required to achieve La_2_O_3_ thermal stability at the target equivalent oxide thickness (EOT). The effects of process variations on *V_th_*, *I_on_* and *I_off_* were investigated. The improved voltage scaling resulting from the lower *V_th_* value is associated with the increased *I_off_* due to the improved drain-induced barrier lowering as the gate length decreases. The performance of the 14-nm planar bulk PMOS is comparable to the performance of the FDSOI and FinFET technologies at the same gate length. The comparisons made with ITRS, the International Roadmap for Devices and Systems (IRDS), and the simulated and experimental data show good agreement and thus prove the validity of the developed model for PMOSs. Based on the results demonstrated, planar PMOSs could be a feasible alternative to FDSOI and FinFET in balancing the trade-off between performance and cost in the 14-nm process.

## 1. Introduction

Great strides in the microelectronics industry have underpinned the development of complementary metal-oxide-semiconductor (CMOS) technology in recent decades. This has contributed to the wide availability of personal computing devices at present and formed the basis of the digital revolution. The ability to continually provide enhanced functionality and reduced power and cost is essential to evolving new technologies driving the development of the Internet of Things (IoT) [1]. In the past few decades, the scaling down of Si metal-oxide-semiconductor field-effect transistors (MOSFETs) to smaller dimensions has been the key driving force of technological growth in the semiconductor and electronics industries. Scaling down planar CMOSs has significant challenges in addressing short-channel effects (SCEs) and voltage roll-off, which degrades the on/off current ratio (*I_on_*/*I_off_*) due to higher *I_off_* values [2]. MOSFETs have steadily been scaled down over the years, resulting in modifications [3]. Developments such as high-k/metal gate technology were introduced and widely adopted as devices were scaled down lower than the 45-nm node [4]. Traditionally, the technology node refers to the dimension of gate length, in which smaller transistors are faster and more power-efficient. In recent years, the technology node has become the commercial name for marketing purposes, referring to a specific generation of chips made using a particular technology. It does not correspond to any gate length and there are discrepancies among foundries in terms of the process’s node name. At the 5-nm technology node, comparisons have been made of transistor performance between FinFET and gate-all-around (GAA) technologies with actual gate lengths of 16 nm [5]. In regard to 16-nm CMOS technology, comparative studies of lifetime reliability between planar MOSFETs with a gate length of 30 nm and FinFETs with a gate length of 20 nm have been undertaken [6].

Gate leakage is minimized through the use of high-k dielectrics, allowing for more gate oxide thickness scaling. The use of a metal gate (rather than a polysilicon one) prevents poly depletion, eliminates the *V_th_* pinning problem that occurs with poly on high-k dielectrics, and screens soft optical phonons for better mobility [7,8]. The high-k dielectric approach increases the k-value, contributing to the gate capacitance and reducing the gate leakage compared to conventional SiO_2_ devices. A high-k material will have a smaller EOT than SiO_2_ and achieve similar capacitance using a thinner material. The concept of EOT is shown in (1).

(1) $$\mathrm{EOT}=\left(\frac{k_{SiO_{2}}}{k_{high-k}}\right)t_{high-k}$$

where $t_{high-k}$ is the high-k material’s thickness, and $k_{SiO_{2}}$ and $k_{high-k}$ are the dielectric constants of SiO_2_ and the high-k material, respectively. However, even with high-k dielectrics, scaling planar bulk MOSFETs below the 20-nm node has proven difficult because the gate dielectric EOT cannot be scaled according to Dennard’s scaling laws. Beyond the 22-nm node, these changes in conventional Si MOSFET planar bulk architecture were insufficient to achieve the performance metrics indicated by the ITRS specifications. Therefore, researchers have been exploring alternative advanced transistor architectures such as multigate, GAA [9], fully depleted silicon-on-insulator (FDSOI) [10,11] and fin field effect transistor (FinFET) [12,13] devices. Apart from process integration problems, another major challenge is to monitor process variations for such small-geometry devices so that the statistical variations of system parameters, such as *I_on_* and *V_th_*, are limited within acceptable limits. New device architectures such as FDSOI and FinFET are known to mitigate these effects and improve electrostatic control channels. The FDSOI transistor is a planar device of which the channel consists of a thin (shallow) silicon layer such that the gate electrode is able to exercise complete electrostatic control. Both FDSOI and FinFET were introduced to solve the significant SCE problems in bulk planar transistors for process nodes below 22 nm.

**Figure 1:**
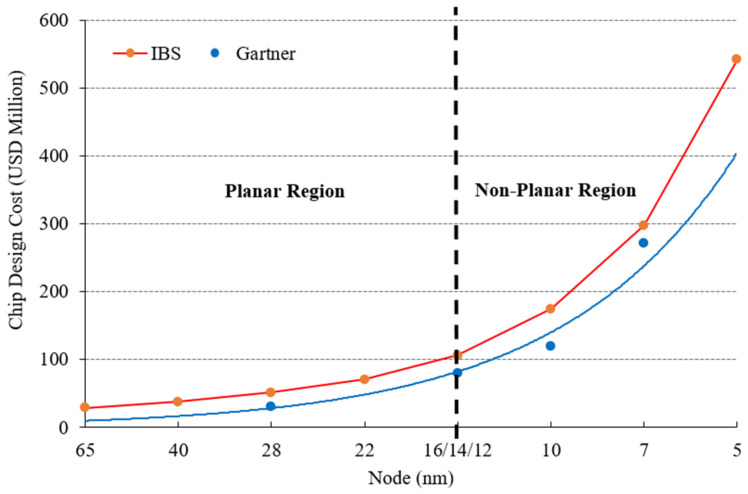
Chip design costs as the technology node advances.

As FDSOI and FinFET continue to garner interest as innovations in silicon process technologies, not many studies have been performed on planar bulk MOSFET technology below 28 nm. According to the Center for Security and Emerging Technology (CSET), a think tank on national and international emerging technologies based in the United States, 3-D transistors such as multi-gate field-effect transistors (MuGFETs), FinFET, and GAAFETs are used primarily in chips for advanced artificial intelligence (AI) applications at technology nodes ≤16 nm for competitive performance. However, planar transistors are still very commonly used for chips at less advanced nodes (>20 nm) [14]. As depicted in Figure 1, chip design and manufacturing costs increase exponentially as technology nodes advance and when changing from transistors with a planar architecture to those with a non-planar architecture. In a rough comparison, Figure 1 shows that the chip design cost for a planar region is around USD 1.6 million per node, whereas for a non-planar region it is about USD 40 million per node (nm) [15]. It is not surprising that only 8.5% of global fabrication capacity is able to be used to fabricate advanced AI chips at ≤16 nm. In comparison, only a fraction of this 8.5% is currently used for this purpose [15]. Therefore, the 28-nm planar node still commands a significant market size in several applications such as artificial intelligence (AI), IoT/edge computing, radio frequency (RF), and wearables. Currently, the Taiwan Semiconductor Manufacturing Company (TSMC) and the United Microelectronics Corporation (UMC) offer a 22-nm planar bulk process, whereas GlobalFoundries is shipping a 22-nm FDSOI [16]. China’s semiconductor industry has predicted that it will have 28-nm chips as its main choice for a long time [17], and technologies for the further downscaling of planar transistors are crucial for the long-term sustainability of semiconductor industries.

Many mid-tier mobile phone applications do not need the highest performance, but they are also susceptible to power efficiency issues. Similarly, many IoT applications require very little raw computing power, but they need to reduce power consumption significantly. Many companies are mulling the idea of moving to 16 nm/14 nm and beyond 28 nm [18]. Equivalent oxide thickness (EOT) scaling with a high-k/metal gate approach has demonstrated the ability to show good performance using a gate-first process, as reported by Chen [19]. Many studies have been performed on the use of 28-nm planar bulk MOSFETs [20] to overcome scaling challenges from 65-nm to 28-nm [21,22]. The statistical optimization of the modelling of process parameters and the effects of process parameter variability for *V_th_* has been conducted by researchers [23,24]. A 28-nm planar bulk CMOS has shown better homogeneous and heterogeneous integration capability in deep sub-micrometer CMOS applications, with good efficiency and low noise levels, while exhibiting better reliability and robustness than a 16-nm FinFET [25]. FinFET processes exhibit more self-heating effects than planar devices due to their higher thermal resistance, causing reliability issues and inconsistent *V_th_* behavior [26]. FinFETs and today’s high-k/metal gate (HKMG) technologies are based on the same underlying mobile driving force.

However, FinFETs are susceptible to self-heating and are more expensive than traditional planar transistors. Comparative studies investigating the effects of bias temperature instability on lifetime reliability indicate trade-offs between planar MOSFETs and FinFETs in advanced nodes such as 16 nm [6]. Therefore, planar bulk MOSFET technology using mature and conventional processing technologies can be an alternative manufacturing option compared to the FDSOI and FinFET approaches beyond 28 nm, if unwanted SCEs can be suppressed [27]. The 28-nm node is a mature process technology that has seen increased utilization in applications such as over-the-top (OTT) boxes, smart televisions, organic light-emitting diode (OLED) drivers, connectivity chips, 4G transceivers, and edge computing. However, they have suffered a decline in the area of smartphone application processors, in which high-end chipsets are usually used. The 28-nm process technology offers cost-competitiveness by balancing reliability and performance. They will see strong demand in the next five years, as more emerging applications and smart IoT devices gain popularity [17]. In the 22-nm node, TSMC and UMC are already offering 22-nm planar bulk processes [16]. Downscaling methods for planar devices may gain further attention from the industry beyond the 22-nm scale in order to achieve a power/performance/cost balance.

In this paper, a 14-nm-gate-length (*L_g_*) PMOS with a direct high-k/Si structure has been realized via virtual fabrication using SILVACO software. SCEs [28] are influenced by both the EOT and physical gate oxide thickness, so it is essential to pay attention to both parameters in order to decrease the gate’s leakage to an acceptable level. Increased attention has been paid to La_2_O_3_ gate dielectrics and much research has been carried out to examine their proper functioning [29,30,31,32]. The form thickness mainly determines the structural properties of La_2_O_3_ and the annealing temperature has to be well controlled to ensure the stability of the La_2_O_3_ layer [33,34]. Therefore, the *I_on_*/*I_off_* ratio is also essential area of focus, in addition to concentrating on the *I_off_*. However, with a lower supply voltage, *I_on_*/*I_off_* decreases dramatically. Thus, the ability to suppress *I_off_* but to increase *I_on_* at the same time will enhance gate controllability and the overall performance of the transistor. The Taguchi L27 optimization method, focusing on the *V_th_* and *I_off_* values, is used herein to enhance the planar bulk MOSFET design.

## 2. Materials and Methods

**Figure 2:**
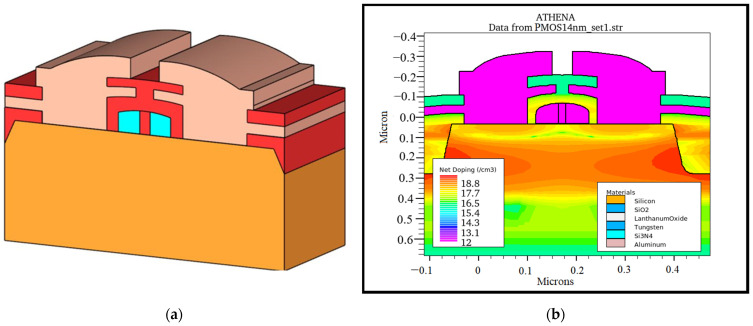
(**a**) Structure of 14-nm-gate-length PMOS; (**b**) load profile of 14-nm bulk PMOS.

A 14-nm La_2_O_3_-based PMOS was fabricated virtually using advanced-process simulation tools from SILVACO TCAD software, version 2020, by SILVACO International, Santa Clara, CA, USA. The design of the PMOS with high-k/metal gate (HKMG) technology was modelled based on previous research [35,36] and simulated using the ATHENA process simulator [37]. La_2_O_3_ was identified to be the high-k oxide and tungsten as the metal gate for the fabricated PMOS [38,39]. The 3D schematic structure of the PMOS is shown in Figure 2a,b shows the PMOS load profile with the input parameters’ net doping concentrations. This process followed by halo implantation to adjust the *V_th_* value to meet ITRS requirements [40].

The PMOS fabrication design and processes were based on previous experiments using high-k/metal technology [41,42]. The data used in the design of the 14-nm PMOS fabrication are summarized in Table 1. Variables such as temperature, time, and material were altered to bring *V_th_*, *I_off_,* and other parameters into an acceptable range based on mature semiconductor device fabrication methods [43] during the simulation. Equation (2) describes the *V_th_* for short-channel PMOSs [44].

(2) $$V_{th}=V_{fb}-2\varphi_{B}-F_{1}\frac{\sqrt{2\varepsilon_{si}qN_{a}\left|\left(-2\varphi_{B}+V_{sb}\right)\right|}}{C_{ox}}$$

where $N_{a}$ is the doping density in the silicon under the MOS gate, $V_{fb}$ is the gate voltage required to compensate for work function differences between the gate and substrate, $V_{sb}$ is the substrate voltage, $\varphi_{B}=\left(KT/q\right)ln\left(N_{a}/n_{i}\right)$ is the position of the Fermi level in the bulk material with respect to the intrinsic level, $\varepsilon_{si}$ is the permittivity of silicon, $C_{ox}$ is the oxide capacitance, and q is the elementary charge. The charge-sharing factor $F_{1}$ is always less than that of short-channel devices and approaches unity for long-channel devices.

In the linear extrapolation method, *V_th_* is extracted by calculating the *I_d_-V_g_* curve’s maximum slope, finding the intercept with the *x*-axis, and subtracting half the drain voltage value from the intercept. The *V_th_* of the PMOS is determined by looking for the voltage where the *I_d_* reaches a user-defined value. *I_d_* is larger for a short-channel PMOS because *V_th_* is less than that of a long-channel PMOS due to charge-sharing and drain-induced barrier lowering (DIBL). *I_d_* is given by

(3) $$I_{d}=\frac{1}{2}\mu_{p}C_{ox}\cdot \frac{W}{L}{\left(V_{gs}-V_{th}\right)}^{2}\left(1+\lambda V_{ds}\right)$$

where *μ_p_* is mobility, W is the transistor’s width, L is the length of the transistor, *V_gs_* is the gate-source voltage, *V_ds_* is the drain-source voltage, and λ is the channel length modulation coefficient.

The sub-threshold current (*I_sub_*) is the current between the drain and the source when the transistor is off, and is also the most dominant *I_off_* component. The *I_sub_* in a short-channel MOSFET can be expressed as in Equation (4) [45],

(4) $$I_{sub}=I_{o}\left[1-exp\left(\frac{V_{sd}}{V_{th}}\right)\right]\cdot exp\left(\frac{V_{sg}-\left|V_{th}\right|-V_{off\prime }}{nV_{T}}\right)$$

(5) $$I_{o}=-\mu \frac{W}{L}\sqrt{\frac{q\varepsilon_{si}N_{D}}{2\varphi_{S}}}\cdot V_{T}^{2}$$

where n is the subthreshold slope factor, $I_{o}$ is the process-dependent parameter, and $V_{T}$ is the thermal voltage.

**Figure 3:**
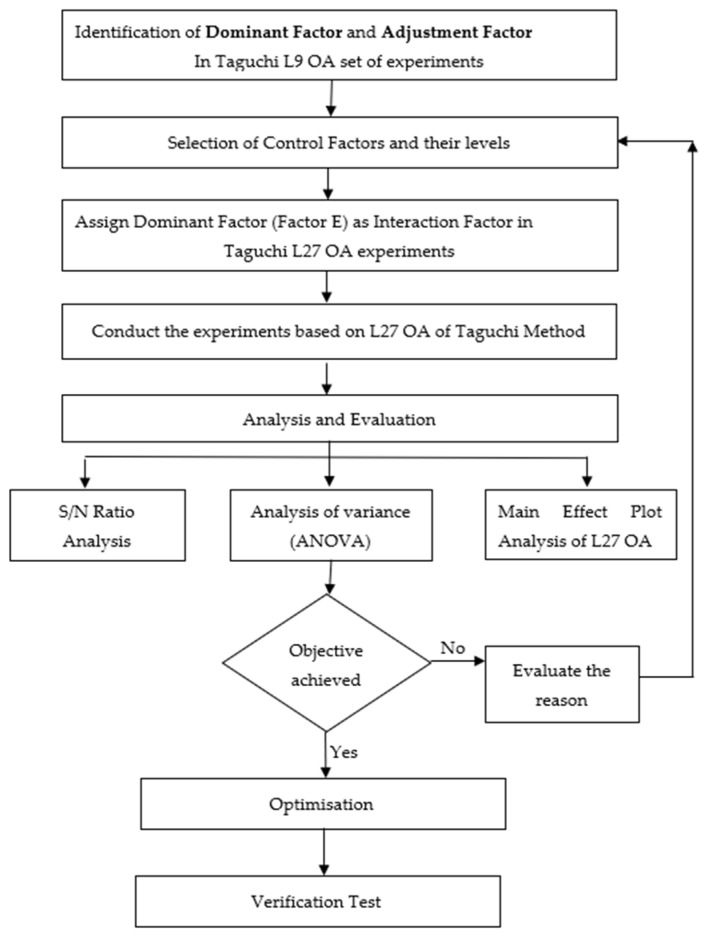
Taguchi analysis and ANOVA.

The PMOS was first designed using the Athena module and was electrically characterized using the ATLAS module from Silvaco TCAD tools. The important parameters were then extracted for the optimization of process parameters using a Taguchi L9 orthogonal array (OA). The L9 OA of the Taguchi method was employed for the optimization process to determine the factors that influence the transistor performance. The determination of the signal-to-noise ratio (*S/N*), analysis of variance (ANOVA), main effects plot analysis, and optimum levels for *V_th_* and *I_off_* were based on the results of the L9 OA experimental run. Dominant and adjustment factors were determined based on this process and verification tests were carried out to validate the optimal levels selected for the process parameters of the L9 OA. Figure 3 shows a flow chart of the process parameter design and optimization of a 14-nm planar PMOS.

The Taguchi method was used to optimize the *V_th_* of the designed PMOS. The dominant factor identified based on the Taguchi analysis of the L9 OA was denoted as Factor E, which was the interaction factor under investigation. Four control factors were selected, which decided the important design parameters of the PMOS from sets of experiments conducted using the L9 OA. Three levels were considered for each variable control factor, as depicted in Table 2. The table depicts the process parameters and their levels, selected based on the Taguchi analysis of the L27 OA. Among all the process parameters, these five process parameters have the most impact on the electrical characteristics of the device.

The L27 orthogonal array analyses of output responses *V_th_*, *I_on_*, and *I_off_* were simulated and recorded. After retrieving the results for *V_th_*, *I_on_*, and *I_off_*, the process parameters of the PMOS device were then statistically modelled using the Taguchi method. The Taguchi method was employed to analyze the *V_th_* value using *S/N* ratio analysis of the nominal-the-best value, *(S/N)_NTB_* [46]

(6) $${\left(\frac{S}{N}\right)}_{NTB}=-10\;log\left(\frac{\mu^{2}}{\sigma^{2}}\right)$$

where *µ* is the mean and *σ* is the variance. The *I_on_* of the device is optimized using *S/N* ratio analysis of the larger-the-better value, *(S/N)_LTB_* [46]

(7) $${\left(\frac{S}{N}\right)}_{LTB}=-10\;log\frac{1}{n}\left(\overset{n}{\underset{i=1}{\sum }}\frac{1}{y_{i}^{2}}\right)$$

where *n* is the number of tests and $y_{i}$ is the experimental value of *I_on_*, whereas the *I_off_* of the device is optimized using *S/N* ratio analysis of the smaller-the-better value, *(S/N)_STB_* as expressed by [46]

(8) $${\left(\frac{S}{N}\right)}_{STB}=-10\;log\frac{1}{n}\left(\overset{n}{\underset{i=1}{\sum }}y_{i}^{2}\right)$$

where *n* is the number of tests and $y_{i}$ is the experimental value of *I_off_*.

The L27 OA was adopted for four control factors with three factor levels. The trial runs were carried out according to the L27 OA and the simulated values of *V_th_* with respect to the different levels of the experiment and their respective factor levels, derived from Table 2. Through the ANOVA, we aimed to statistically analyze the variance caused by each factor in relation to the overall variation in the results. By performing this ANOVA, the percentage contributions, using the formulas of each factor, were identified.

The optimization of the transistors was carried out by adjusting the process parameters using the Taguchi method. The halo implantation dose, halo implantation energy, source-drain implantation dose, source-drain implantation tilt, and compensation implantation were the selected parameters in this analysis. Sacrificial oxide layer temperature and BPSG temperature were noise influences, on the other hand. The Taguchi method of experimental design was used to establish relationships between the various control factors in this study. The proposed method used a statistical approach based on the Taguchi method and the analysis of variance (ANOVA) technique to assess the effect of each parameter. Taguchi’s design approach is an effective tool for improving a process’s performance characteristics. A Taguchi design experiment aims to identify and design the process settings and parameters that are least sensitive to noise influences.

## 3. Results

### 3.1. Virtual Fabrication of 14-nm-Gate-Length PMOS

**Figure 4:**
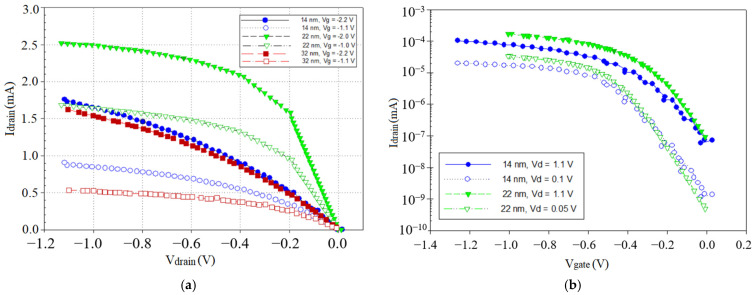
(**a**) Output characteristics of 14-nm-, 22-nm-, and 32-nm-gate-length planar bulk PMOSs; (**b**) transfer characteristics of 14-nm- and 22-nm-gate-length planar bulk PMOSs.

The PMOS device was simulated by initially forming a p-type silicon substrate with phosphorus doping of 4.5 × 10^11^ cm^−2^. Then, the diffusion process was carried out to grow the oxide. The process continued with the implantation of boron difluoride (BF_2_), at a dose of 1.8 × 10^11^ cm^−2^. After that, the metal gate was deposited to form the gate on top of the high-k material, La_2_O_3_. Figure 4a shows the characteristic curve between the drain current (*I_d_*) and drain voltage (*V_d_*) at different gate voltages (*V_g_*) of 1.0 V, 1.1 V, 2.0 V, and 2.2 V, whereas Figure 4b shows the plot of *I_d_* versus *V_g_* at *V_d_* = 0.1 V and 1.1 V, and *V_d_* = 0.05 V and 1.1 V, respectively, for the 14-nm and 22-nm devices [35,47]. *V_th_* was calculated by subtracting half of the applied drain bias from the overall slope of the *I_d_-V_g_* curve and determining the intercept with the *x*-axis.

*I_on_* and *I_off_* values were extracted from the sub-threshold graph, as shown in Figure 4b. The results revealed significant increases in *I_d_* by scaling the *L_g_* down to 14 nm (Figure 4a) compared with 32 nm, although these were less than those in the 22-nm PMOS. The drain current showed acceptable behavior due to the length reduction, and the current values for the 14-nm PMOS in the range of measurements were significantly better than those of the 22-nm PMOS. The effects of downscaling on the sub-threshold leakage current are shown in Figure 4b, where the *I_d_-V_g_* graph is shown for high-k/metal gate PMOSs with different *L_g_* values. The appropriate work function of the metal gate is crucial in order to control the *V_th_* value and to improve SCEs on device performance [48,49]. By incorporating La_2_O_3_ dielectrics into a PMOS with a matching metal gate, the device’s performance is further enhanced and improved with optimization. One of the prominent features of the high-k/metal gate system is Fermi level pinning, associated with oxygen vacancy. La_2_O_3_ high-k material exhibits ionic binding, and it is essential to have precise control over the oxygen vacancy in the material and process technology. The subthreshold behavior of the device can be reasonably controlled through the proper adjustment of the metal gate work function. The levels of the sub-threshold leakage current for the 14-nm PMOS with the different channel lengths were in the order of 10^−7^ A, as predicted by the ITRS. The *I_on_* for the 14-nm PMOS was calculated to be 10,900 µA/µm and the *I_off_* was 8.06 × 10^−8^ A/µm.

### 3.2. Taguchi L9 and L27 Orthogonal Array Method

**Figure 5:**
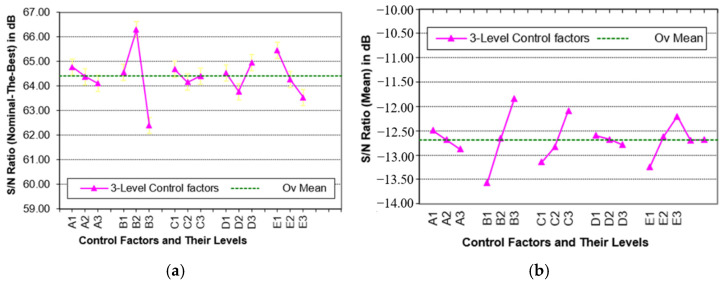
(**a**) Factor effects plot of *(S/N)_NTB_* for *V_th_*; (**b**) factor effects plot of *(S/N)_Mean_* for *V_th_*.

The dominant factor identified based on the Taguchi L9 OA was denoted as Factor E, referring to the interaction factor under investigation. Two noise factors at two levels, namely, the annealing temperature of the sacrificial oxide layer (X1 = 900 °C, X2 = 902 °C) and boron phosphor silicate glass (Y1 = 850 °C, Y2 = 852 °C) were included in the Taguchi analysis. Generally, it was found that the source/drain implantation dose is a dominant factor and significantly affects *V_th_* in L9 simulations. Based on the results of the Taguchi L9 runs, the source/drain implantation dose was identified as Factor E to be used for the design of the experimental matrix using Taguchi’s L27 OA technique. A final judgement was made to utilize four input parameters with three levels each. A total of 3 × 3 × 3 × 3 = 81 runs were required in the experiment for four input parameters. The Taguchi method was utilized to optimize the process parameter variations on the *V_th_* of the PMOS device. The Taguchi method uses an OA with twenty-seven rows of experimental data. The Taguchi method has the capability of selecting the best level’s combination of process parameters with a smaller number of experiments. Table 3 shows the *S/N* ratio of the process parameters and factor effects for the PMOS device, whereas Table 4 shows the final results of the confirmation run for *V_th_*, *I_off_*_,_ and *I_on_* with different combinations of noise factors. The key values of the 14-nm-gate-length PMOS before and after optimization are compared with the ITRS 2013 targeted values. Figure 5a,b show the factor effect graphs for *(S/N)_NTB_* and *(S/N)_Mean_* used to determine the control factors which give the most significant effects. Referring to the graphs, the highest *S/N* ratio of each process parameter level, to achieve nominal *V_th_*, can be discovered.

*S/N* ratios were calculated for each factor and level; they were tabulated as shown in Table 3. The *S/N* ratio and mean were graphed and used to identify the important factors. The *(S/N)_NTB_* graph and the *(S/N)_Mean_* graph for *V_th_* are shown in Figure 5a,b, respectively. Factor B has a large effect on *V_th_* and a small effect on the mean, according to the response graph. Factor C has a relatively small effect on *V_th_*. Since the quality characteristics type was *NTB*, the response variable was based on the *S/N* ratio, in terms of the standard deviations and mean outputs. The significance of any of these factors was determined by identifying the effect and ranking of the mean and *S/N* ratio for each controllable factor. All factors were then ranked. Based on the response shown in Table 3, the highest value of each *S/N* ratio for each factor was identified, along with the dominant factor. The highest value of each *S/N* ratio for each factor was used to form the optimum combination of levels of each factor. The larger the delta (Δ) value for a parameter, the larger the effect the variable has on the process. This implies that factors B, E, and D were more important than the rest of the factors. It can be observed that the same change in signal causes a larger effect on the output variable being measured.

## 4. Discussion

The scaling of FDSOI technology to the 14-nm scale was demonstrated in [50]. The ability to control the back-plane potential of both transistor types to modulate (and particularly to lower) the *V_th_* value, which improves the possibility of advanced dynamic *V_th_* tuning, makes this significantly different from bulk technology [51]. One major advantage of FDSOI over bulk technology is the usage of back-gate biasing schemes to control *V_th_* [51].

Table 5 and Table 6 show the data from the latest ITRS and IRDS editions for planar bulk, FDSOI, and FinFET devices for reference in regard to different technology nodes vs. their physical gate lengths. IRDS has been widely accepted as the successor to the final ITRS roadmap presented in 2016 and the latest process nodes are simply a commercial name and are no longer based on transistors’ gate-lengths, as was the case in the 1960s up to the late 1990s. Over the years, the electronic industry’s structure and requirements have evolved beyond the requirements of the semiconductor industry, which led to the end of the ITRS roadmap. IRDS presents integrated system requirements with device requirements to ensure the continued evolution of computing. In order to achieve the parameter values projected in the roadmap, *V_th_* and *I_off_* targets were set and the initial set of parameters was chosen based on other reported works, scaling rules, and device principles. It was observed in the optimized results that a 14-nm-gate-length bulk PMOS incorporating a La_2_O_3_ dielectric in the gate could meet the *I_off_* target identified by the ITRS and enhance *I_on_* up to one order of magnitude higher than the target.

As indicated in Table 5, the results for our 14-nm gate-length bulk PMOS incorporating the La_2_O_3_ dielectric after optimization showed a 24.1% improvement in the *I_on_/I_off_* ratio. Thus, the optimized PMOS device showed a higher switching speed (due to higher *I_on_/I_off_*) and low power dissipation (due to lower *I_off_*) for circuit applications. The planar bulk PMOS showed an *I_off_* of 10^−8^ A and an *I_on_* of 10^−2^ A, with an *I_on_/I_off_* ratio of around 10^5^ for a drain bias of 1.1 V. The usage of a high-k/metal gate design is important in order to enhance channel control and improve performance. In work reported by Wang et al. [52], a high-performance 25-nm-gate-length planar PMOS with a HfO_2_ gate dielectric was fabricated in a controlled environment with a good process integration scheme. Table 6 displays the experimental and simulation values for FDSOI and FinFET, respectively. FDSOI and FinFET have higher *I_on_* and lower *I_off_* values compared to the planar PMOS at the same gate length. The evolution from planar PMOSs to more complex designs occurred in order to keep *I_off_* under control and to achieve better performance. The key reason for moving to FinFET below the 28-nm gate length was the excessive *I_off_* exhibited by planar devices, and FinFET architecture is observed to display better SCE suppression, lower switching times, and higher current density. However, it is considerably difficult to control the dynamic *V_th_* for FinFET, leading to high capacitances, which involves high fabrication costs.

The *I_on_/I_off_* ratio is an important figure of merit to evaluate device performance (higher *I_on_*) and low leakage power (lower *I_off_*). *I_on_/I_off_* is observed to be on the order of 10^4^, which is close to the minimum requirement for modern digital circuits and minimizes static power consumption mainly due to leakages. The results showed that it is effective to use thin gate oxide and a high-k dielectric material to suppress process-variation-induced *V_th_* fluctuations. As the EOT is lowered, the size of the potential barrier for planar MOSFETs is reduced. The subthreshold slope (SS) is a measure of effective gate control, and its reliance on the variability source is critical when evaluating quiescent leakage currents. The SS is extracted from an ensemble of transfer characteristics at various *V_d_* values, ranging from 0.1 V to 1.0 V in the subthreshold region. Channel mobility in high-k/metal gate PMOSs can be further enhanced through channel strain engineering, in which compressive strain for PMOSs is used to improve the performance of high-k/metal gate transistors. In addition, metal-gate electrodes with the correct matching work functions are required to achieve the satisfactory *V_th_* in PMOSs. Effective work function engineering is a critical success factor in controlling SCEs for planar PMOSs.

Planar devices are a mature technology with conventional processing techniques and established production technologies in information communication technology (ICT), and are integrable with conventional materials such as Si. These premises give viable, practical, and cost-efficient options as a consideration for integrated circuit (IC) manufacturers to explore for IoT devices in the immediate future and in markets such as smart wearables that demand portability, integrability, connectivity, and price sensitivity. Our results show that 14-nm planar MOSFETs exhibit good performance, comparable to some sub-10-nm-gate-length regimes. Recent technology nodes below 22 nm refer to a specific generation of ICs made within a particular technology and do not correspond to any gate length or half pitch. New technology nodes are introduced to achieve overall gains in system performance, which require optimization of the technology, circuit, packaging, and architecture to balance performance, cost, and development time.

## 5. Conclusions

In conclusion, a solution for achieving an optimum *V_th_* value was successfully predicted through the combination of process simulation, device simulation, and the use of an L27 OA as part of the Taguchi method. These tools have the ability to predict the device’s process recipe. The *V_th_* and *I_off_* responses were the primary focus of this study. This was regarded as the main factor in assessing the functionality of a PMOS device. The L27 OA within the Taguchi method was used to investigate the primary effects of various factors (process parameters) on the *V_th_* value. The 14-nm PMOS had an *I_off_* of 10^−8^ A and an *I_on_* of 10^−2^ A with an *I_on_*/*I_off_* ratio of around 10^5^ after optimization using the Taguchi method. For many analog and digital applications in a cost-sensitive market, product requirements demand the careful consideration and selection of a suitable design, whether planar bulk, FDSOI, or FinFET, as well as trade-offs in terms of performance, power efficiency, and leakage. Although FinFET has captured the top segment of the mobile market with advanced technology nodes, planar CMOS technology offers the advantage of the easier integration of IoT devices with analog and RF parts. As planar transistors exhibit many new challenges beyond 28 nm, many researchers have turned to alternative designs such as FDSOI and FinFET as the race for advanced high-performance devices continues. However, the use of a 14-nm-gate-length planar PMOS with good performance parameters offers an option for digital applications in a cost-competitive market.

## Figures and Tables

**Table 1:** PMOS fabrication design.

Process Step	PMOS Parameters	Process Environment
Silicon substrate	<100> orientation	
Retrograde well implantation	200 Å oxide screen	970 °C, 20 min of dry O_2_
		4.5 × 10^11^ cm^−3^ phosphorus
		30 min, 900 °C diffused in nitrogen
		36 min, dry O_2_
STI isolation	130 Å stress buffer	900 °C, 25 min of dry O_2_
		1500 Å Si_3_N_4_, applying LPCVD
		1.0 μm photoresist deposition
		15 min annealing at 900 °C
Gate oxide	Diffused dry O_2_	0.1 min, 815 °C
*V_th_* adjust implant	1.8 × 10^11^ cm^−2^ BF_2_	5 keV implant energy, 7° tilt
		20 min annealing at 800 °C
High-k/metal gate deposition	0.002 μm La_2_O_3_	17 min, 900 °C annealing
	0.0038 μm W	
LDD implantation	3 × 10^13^ cm^−3^ phosphor	20° tilt
Sidewall spacer deposition	0.008 μm Si_3_N_4_	
S/D implantation	1.4 × 10^13^ cm^−3^ Boron	10 keV implant energy
		7° tilt
PMD deposition	0.3 μm BPSG	25 min, 850 °C annealing
Metal 1	0.04 μm aluminum	
IMD deposition	0.04 μm BPSG	15 min, 950 °C annealing
Metal 2	0.12 μm aluminum	

**Table 2:** Process parameters and their levels.

Sym.	Control Factor	Units	Level 1	Level 2	Level 3
A	Halo Implantation Dose	atom/cm^3^	5.35 × 10^13^	5.40 × 10^13^	5.45 × 10^13^
B	Halo Implantation Energy	keV	158	160	162
C	Source/Drain Implantation Tilt	°	6.5	7.0	7.5
D	Compensation Implantation	atom/cm^3^	1.0 × 10^12^	1.1 × 10^12^	1.2 × 10^12^
E	Source/Drain Implantation Dose	atom/cm^3^	1.38 × 10^13^	1.41 × 10^13^	1.43 × 10^13^

**Table 3:** *S/N* ratios of process parameters and factor effects for a PMOS device.

Sym.	Level 1	Level 2	Level 3	Factor Effect Nominal (%)	Factor Effect Mean (%)
A	64.77	64.38	64.10	2.10	2.76
B	64.54	66.29	62.41	69.72	55.00
C	64.68	64.16	64.40	1.28	21.60
D	64.52	63.77	64.95	6.52	0.69
E	65.45	64.26	63.53	17.18	19.95

**Table 4:** Final results of the confirmation run for *V_th_*, *I_on_* and *I_off_*.

A1B2C3D3E1	X1, Y1	X1, Y2	X2, Y1	X2, Y2	Mean
*V_th_* (V)	−0.233449	−0.233613	−0.233489	−0.233653	−0.233551
*I_on_*(µA/µm)	10,900	10,900	10,860	10,900	10,900
*I_off_*(nA/µm)	107	108	107	108	80.6

**Table 5:** Comparison of different gate-length planar bulk PMOS values vs. ITRS.

Planar Bulk PMOS (Experimental)							Planar Bulk (ITRS2013)		This Result	
Node Range	32	65	65	90	100	100	“8/7”	“16/14”	Before Optimisation	Optimised Results
*L_g_*	25 nm	30 nm	33 nm	35 nm	45 nm	50 nm	14 nm	20 nm	14 nm	14 nm
*V_th_* (V)	N/A	N/A	−0.2	N/A	−0.25	−0.2	0.230	0.190	−0.230781	−0.23355
*I_on_*(µA/µm)	488	285 at 0.85 V	400 at 1.2 V	272 at 0.85 V	1070 at 1 V	700 at 1.2 V	>1267	>1348	10,900	10,900
*I_off_*(nA/µm)	0.83	<83 at 0.85 V	300 at 1.2 V	100 at 0.85 V	100 at 1 V	40 at 1.2 V	<100	<100	100	80.6
*I_on_/I_off_*	587,951	3433.735	1333.333	2720	10,700	17,500	12,670	134,800	109,000	135,236
*SS* (mV/dec)	85	100 at 0.85 V	N/A	92.3 at 0.85 V	N/A	N/A	N/A	N/A	98.43	76.98
*DIBL* (mV/V)	77	100	N/A	N/A	N/A	N/A	N/A	N/A	71.2	68.57
Ref	[52]	[53]	[54]	[55]	[56]	[57]	[58]			

Columns highlighted in gray show a direct comparison of FDSOI and FinFET with planar MOSFET at *L_g_* = 14 nm.

**Table 6:** Comparison of different gate-length FDSOI and FinFET values.

	FDSOI			FDSOI (IRDS 2017)	FinFET			FinFET (IRDS 2020)	
Node Range	“9/8”	“11/10”	“16/14”	“10”	“3”	“3”	“16/14”	“5”	“3”
*L_g_*	14 nm ^1^	18 nm	25 nm	20 nm	12 nm ^1^	14 nm	30 nm	18 nm	16 nm
*V_th_* (V)	N/A	−0.2	−0.3	0.190	−0.28	N/A	−0.24	0.222	0.237
*I_on_*(µA/µm)	1014 at 1.0 V	350 at 1.0 V	550 at 0.9 V	>972	1500 at 0.6 V	N/A	292 at 0.8 V	>854	>912
*I_off_*(nA/um)	16 at 1.0 V	100 at 1.0 V	3 at 0.9 V	<10	10 at 0.6 V	N/A	7.5 at 0.8 V	<10	<10
*I_on_/I_off_*	63,375	3500	183,333	N/A	150,000	N/A	38,933	N/A	N/A
*SS* (mV/dec)	N/A	75 at 1.0 V	80 at 0.9 V	68	70 at 0.6 V	80 at 0.3 V	72 at 0.8 V	78	82
*DIBL* (mV/V)	N/A	75	85	N/A	N/A	75	50	N/A	N/A
Ref	[59]	[60]	[61]	[62]	[63]	[64]	[65]	[66]	

^1^ simulation; columns highlighted in gray show a direct comparison of FDSOI and FinFET with planar MOSFET at *L_g_* = 14 nm.

## Data Availability

The data presented in this study are available on request from the corresponding author.
